# Hospital Meetings

**Published:** 1895-12-14

**Authors:** 


### HOSPITAL MEETINGS.

### SURGICAL AID SOCIETY.

The Lord Mayor presided on Monday, December 9th, at the thirty-third annual meeting of the Surgical Aid Society, held at Cannon Street Hotel. The report then read showed that the income for the past year had been £10,599, an increase on the amount received for the previous twelve months; 13,345 patients had been helped during that time, and 20,046 surgical instruments distributed. A useful work was also carried on by the loan of invalid chairs, air and water beds, and other appliances to those who could otherwise be quite unable to procure these comforts in illness. The society had lost more than one valuable supporter during 1895, but the committee were glad to report that Mr. John Airey, Mr. Bompas, Q.C., and Sir Francis Jeune had joined their ranks. Contributions received in connection with the meeting amounted to £394.

